# Advances in the Treatment of Autism Spectrum Disorder: Current and Promising Strategies

**DOI:** 10.2174/0109298673252910230920151332

**Published:** 2024-02-06

**Authors:** Konstantin Yenkoyan, Zadik Ounanian, Margarita Mirumyan, Liana Hayrapetyan, Naira Zakaryan, Raisa Sahakyan, Geir Bjørklund

**Affiliations:** 1 Neuroscience Laboratory, Cobrain Center, Yerevan State Medical University after M. Heratsi, Yerevan, Armenia;; 2 Department of Biochemistry, Yerevan State Medical University after M. Heratsi, Yerevan, Armenia;; 3 Department of Radiation Oncology, Inselspital, Bern University Hospital and Department for BioMedical Research (DBMR), University of Bern, Switzerland;; 4 Graduate School for Cellular and Biomedical Sciences, University of Bern, Switzerland;; 5 Department of Research, Council for Nutritional and Environmental Medicine, Mo i Rana, Norway

**Keywords:** Autism spectrum disorder, treatment strategy, management, neuropharmacology, animal model, neurochemistry

## Abstract

Autism spectrum disorder (ASD) is an umbrella term for developmental disorders characterized by social and communication impairments, language difficulties, restricted interests, and repetitive behaviors. Current management approaches for ASD aim to resolve its clinical manifestations based on the type and severity of the disability. Although some medications like risperidone show potential in regulating ASD-associated symptoms, a comprehensive treatment strategy for ASD is yet to be discovered. To date, identifying appropriate therapeutic targets and treatment strategies remains challenging due to the complex pathogenesis associated with ASD. Therefore, a comprehensive approach must be tailored to target the numerous pathogenetic pathways of ASD. From currently viable and basic treatment strategies, this review explores the entire field of advancements in ASD management up to cutting-edge modern scientific research. A novel systematic and personalized treatment approach is suggested, combining the available medications and targeting each symptom accordingly. Herein, summarize and categorize the most appropriate ways of modern ASD management into three distinct categories: current, promising, and prospective strategies.

## 1 INTRODUCTION

Autism spectrum disorder (ASD) is a group of developmental disorders characterized by social and communication impairments, language difficulties, limited interests, and repetitive behaviors. As the reported rates of children with ASD increase, the most recent estimates from the United States by the Centers for Disease Control and Prevention suggest that 1 in every 44 children might be affected [1]. ASD manifests differently depending on the type and severity of the disorder. Moreover, those symptoms are mainly followed by their characteristic comorbidities, such as hyperactivity, seizures, and sensorimotor abnormalities [2]. Because of heterogeneity in clinical manifestations, it is difficult to pinpoint the precise etiology and pathophysiology of ASD, which underlie the core symptoms.

Some of the most urgent tasks researchers face are assessing the various risk factors that can cause ASD, identifying their role in developing autism symptoms, and creating optimal strategies to reduce these symptoms in animals. Factors contributing to the development of ASD are etiologically different and can include genetic mutations, environmental factors, and epigenetics [3], which can further be grouped as genetic versus non-genetic factors. According to various theories, the underlying pathophysiology of ASD involves impairments in neural connectivity [4], neural migration [5], imbalances in excitatory-inhibitory neural activity [6], incorrect synaptogenesis and dendritic morphogenesis [7], disturbances in neuro-immunological disorders, broken mirror neuron theory [8], as well as single gene disorders with autism symptoms [9].

Since most of the aforementioned theoretical approaches cannot be tested and verified in human models, researchers turn to animal models to conduct preclinical studies and to better correlate the numerous etiological pathways of ASD. Such models enable researchers to investigate certain features of the pathophysiology and behavioral variations of ASD in living subjects and to choose the most appropriate model for the study. Knock-out and humanized knock-in mice and rats are generated for many de novo single-gene mutations and copy number variants (CNVs) detected in ASD and comorbid neurodevelopmental disorders to investigate their causes and potential therapeutic approaches [10]. The most appropriate tool for adequately reproducing ASD pathology is the rodent model. Using this approach, researchers can determine and assess the different risk factors of ASD and measure their role in the manifestation of autistic symptoms [11]. Such preclinical studies permit the creation of some of the most rational ways to treat ASD in people or improve patients’ quality of life.

Recent studies on ASD management continue to yield contradictory results, hence why a consensus on ASD treatment strategies is still absent. The list of FDA-approved methods of ASD management is incomplete and includes a scant number of atypical antipsychotics, of which the most effective is risperidone (atypical antipsychotic) [12]. Risperidone does not yield satisfactory results based on patients' overall clinical, behavioral, and lifestyle improvements. Consequently, an increasing number of studies have been focusing on targeting ASD management through the scope of disease pathogenesis, hoping to develop a more robust and diverse approach to this multifaceted disorder.

Henceforth, this review will summarize and categorize the most appropriate ways of modern ASD management based on its diverse pathogenetic routes, subclassifying them into the current (basic), promising, and prospective strategies. Ultimately, a novel systematic and personalized treatment approach is suggested, combining the available medications and targeting each symptom accordingly.

## 2 STRATEGIES FOR ASD TREATMENT

**Fig. (1):**
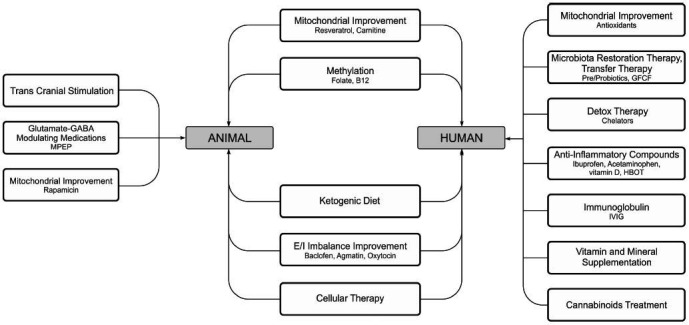
Suggested viable approaches to ASD treatment. Separation of available treatment strategies based on the species and the stage of research: humans (clinical research and practice) and rodents (basic and translational research).

Therapeutic interventions for a complex disorder like ASD need to be multi-directional. Available treatment strategies were divided based on the species and the stage of research: humans (clinical research and practice) and rodents (basic and translational research) (Fig. **1**). No single strategy claims to be a multifaceted solution to the diverse symptomatology of ASD. As such, each one is used for single-target modifications in standalone issues recognized in ASD pathogenesis.

It is suggested to divide treatment strategies in humans into three groups: current, promising, and perspective (Table **1**). Current/basic: These therapies were studied and continue to be verified through expanding research efforts. Most of the strategies in this group are safe and recommended for current clinical use. Promising: Strategies in this group still need to be approved for clinical use but show good signs for the near future after ample research evidence is published and verified. Perspective: Strategies here are considered the cutting-edge of current scientific research. As such, they have a tempting quality to be regarded as complete treatment strategies. However, as studies need to support their safety and viability in clinical practice, they cannot be included in the current recommendations.

Research on treatment strategies that potentially improve the quality of life for people with ASD is ongoing. Newer, safer, and potential aches are still under development. Treatment must be initiated as soon as possible, and specific therapies must be chosen for each case. Herein, treatment strategies undergoing rigorous testing and research are presented. Some are still being tested in animal models to establish their safety levels; others are already approved for testing in human cohorts worldwide.

## 3 IMPROVEMENT OF EXCITATION AND INHIBITION IMBALANCES

Excitation and inhibition imbalances are known targets for ASD treatment. Recent studies highlight many causes for E/I imbalances, such as genetic mutations, disturbances in signaling pathways, and altered neuronal network building. Mutations can occur in scaffolding proteins such as SHANK-3 or synaptic cell adhesion molecules like NLGN3, NLGN4, and NRXN1 [13]. Disturbances in Parvalbumin (PV)-positive inhibitory neurons reduce cortical plasticity and desynchronized gamma oscillations in the hippocampus and neocortex [14]. Altered neuronal network construction results from ASD-associated disturbances in the GABA pathway, which is responsible for synaptic tuning and neuronal wiring [15]. Some genetic findings also support a link between disturbances in GABA-ergic and glutamatergic receptors with ASD [16]. Treatment approaches can be direct, as is the case for agents that act directly on Glutamate / GABA, or indirectly, like correction of mitochondrial dysfunction, improvement of methylation, and restoration of gut microbiota.

Research suggests that zinc (Zn) and copper (Cu) are critical in the GABAergic and glutamatergic systems [17]. About 10% of the total Zn in the brain is found in the synaptic vesicles of glutamatergic neurons. Zinc is known to be involved in regulating GABA and glutamate, particularly through anxiolytic activity and modulating GABAergic inhibition and seizure susceptibility. Studies have also shown that Zn deficiency can impair GABAergic function [17]. Copper is a potent inhibitor of GABA-evoked responses, especially in Purkinje cells. Zinc and Copper may interact with each other and the GABAA receptor complex to modulate synaptic transmission [17].

### 3.1 Dopamine and Serotonin Receptors Antagonist - Risperidone

ASD pharmacotherapy has, so far, been focused on controlling symptomatic behavioral problems like aggression, self-injury, and hyperactivity. Despite showing efficacy in decreasing repetitive movements, temper tantrums, and hyperactivity and improving social interaction, typical antipsychotics are compounded by unwanted extrapyramidal effects (UEE). Atypical antipsychotics seem to show just as much promise in addressing these symptoms without the associated drawbacks of UEEs [18]. In addition, as movement disorders can be an intrinsic part of some disorders on the autism spectrum and be caused by treatment, it is difficult to reliably estimate the efficacy of medications when assessing unusual stereotypies in patients with ASD [18].

Risperidone (Risperdal) is an FDA-approved atypical antipsychotic that exercises promising signs of behavioral modification with a relatively high safety index in patients with bipolar I disorder, psychosis, depression associated with psychosis, schizophrenia (acute and chronic), and autistic disorder [19]. Chemically, risperidone consists of a tetrahydropyrido [1,2-a]pyrimidin-4-one scaffold as the core linked *via* piperidine to a benzisoxazole nucleus (Suzuki *et al.*, 2012) and is specifically designed as a combined receptor antagonist for dopamine D2 and serotonin 5-HT(2A) receptors [20].

Risperidone remains one of the only FDA-approved antipsychotics for the management of ASD-associated irritability in children aged 5 to 16 [21]. Risperidone dosage is based on the weight of patients. Usually, throughout the studies, patients were started on an initial dose that was lower than the target dose, incrementally increased to reach the latter, and then tapered off after a predetermined course of therapy was completed [21-23]. Some studies offer, on average, a supplementary four-week cycle for patients who experienced positive changes from treatment and were willing to continue [24, 25]. The mentioned studies used a slew of approved ASD assessment forms such as the Aberrant Behavior Checklist (ABC), Clinical Global Impression-Severity (CGI-S), Clinical Global Impression-Improvement (CGI-I), Children’s Global Assessment Scale (C-GAS), *etc.* On average, risperidone performed well on most parameters. Experimental groups showed more noticeable and widespread improvements in ASD-associated behaviors and mannerisms. All mentioned studies had patients from both experimental and placebo groups drop out during the study. This may explain why a consensus on a defined pharmacotherapeutic agent for ASD-associated behavioral improvement has not yet been reached.

### 3.2 Glutamate Modulating Medications

Newer treatment approaches connected to glutamatergic neurotransmission are emerging. The high interest in glutamatergic dysfunction in ASD is due to increasing proof implying that it has a crucial role in ASD from the standpoint of peripheral biomarkers, neuroimaging, protein expression, genetics, and animal models. Other glutamate-modulating medications, with approved indications for use in other disorders, are being explored for re-tasking them as treatments for ASD.

The valproic acid animal model of ASD illustrates E/I imbalances caused by the differentiation of glutamatergic neurons and decreased numbers of GABA-ergic neurons [26].

Agmatine is one of the newer candidates to be repurposed for ASD management. It is an endogenous polyamine synthesized from arginine, with a possible role in the pathophysiology of several diseases, including anxiety disorder, depression, and schizophrenia [27, 28]. Agmatine possesses anticonvulsant, antiapoptotic, neuroprotective, antioxidant, anxiolytic, and antidepressant effects [29]. Furthermore, it inhibits the nitric oxide synthase enzyme and exerts antagonist effects on NMDA alpha-2 and imidazoline receptors [29]. According to experimental evidence, agmatine seems therapeutic in ameliorating ASD-like symptoms by modulating E/I imbalance [29]. A single treatment regimen of agmatine normalized the impaired social behaviors alongside hyperactive and repetitive behaviors in the VPA animal model [29]. Agmatine levels are noticeably lower in patients with ASD [29]; thus, low levels might contribute to the pathogenesis of ASD and may serve as a new target for treatment [27].

Another strategy to reduce excitatory neurotransmission is to inhibit metabotropic glutamate 5 (mGlu5) receptors with negative allosteric modulators. The mGlu5 receptor antagonist, 2-methyl-6-(phenylethynyl)-pyridine (MPEP), was assessed in the BTBR mouse model, where an acute treatment regimen decreased repetitive behaviors in BTBR animals (Silverman *et al.* 2012) [30].

### 3.3 NMDA Receptor Modulators

The N-methyl-D-aspartate (NMDA) receptor is a type of glutamate receptor that plays a critical role in regulating synaptic plasticity and is important for learning and memory. Evidence suggests that abnormalities in NMDA receptor signaling may contribute to the development of ASD [31].

Memantine is an NMDA receptor modulator studied as a potential treatment for ASD. It works by blocking the excessive activation of NMDA receptors, which may help to restore the balance between excitation and inhibition in the brain. Several clinical trials have investigated the effectiveness of memantine in treating ASD, and the results have been mixed [32-34]. Other NMDA receptor modulators are also being studied as potential treatments for ASD. For example, d-cycloserine is an NMDA receptor partial agonist that has been shown to improve social behavior and reduce repetitive behaviors in animal models of ASD [35-37]. However, more research is needed to determine whether these findings can be replicated in humans.

The potential of NMDA receptor modulators as a treatment for ASD is still being explored. More research is needed to evaluate their effectiveness and safety in individuals with ASD.

### 3.4 Agonists of GABA Receptors

Agonists of GABA receptors are also considered potential therapeutic agents for ASD [38]. The Fragile X mouse models (Fmr1-knockout) show decreased GABA-ergic input in many regions of the brain and, as a consequence, reduced GABA-ergic input [39].

The selective GABA-B receptor agonist R-baclofen reversed social deficits and decreased repetitive behaviors in a mouse model of Fragile X syndrome [40]. To assess R-baclofen in a larger sample of ASD mouse models, the R-baclofen enantiomer was tested in two inbred strains of mice that demonstrated low sociability and/or highly repetitive or stereotyped behaviors [40]. R-baclofen treatment reversed low scores of repetitive self-grooming and high scores of marble burying in BTBR mice. At non-sedating doses, it reduced the stereotypical jumping behavior in C58/J (C58) mice [40].

In addition, one study included a randomized, double-blind, placebo-controlled trial to test the efficacy of baclofen as an adjuvant to risperidone [41]. Participants were assessed thrice over ten weeks. Their results revealed that participants in the experimental baclofen adjuvant group improved the ABC-C scale more than those with placebo-supplemented risperidone regimens. The improved target ASD-associated behaviors were irritability, lethargy, stereotypic behavior, hyperactivity, and inappropriate speech. Overall, this clinical trial recommends the usage of baclofen as a risperidone adjuvant due to its high safety and efficacy rating [41].

### 3.5 Repetitive Transcranial Magnetic Stimulation

Repetitive transcranial magnetic stimulation (rTMS) is a non-invasive technique that induces excitability and changes in synaptic plasticity in the brain. Using animal models with autistic-like behaviors caused by neonatal isolation, a 2-week low-frequency rTMS treatment effectively improved the acquired autistic-like symptoms in young adult rats. Also, rTMS restored E/I balance at the level of synaptic receptors in synaptosomes by regulating synaptic GABA transmission [42]. rTMS is now actively used in the clinical setup by potentiating inhibitory mechanisms and achieving a significant reduction in the core symptoms of ASD patients [43]. Protocol to study this tool in multicenter randomized controlled clinical trials is already available, pointing out its relevance [44].

### 3.6 Treatment with Oxytocin (OXT)

Central OXT deficit is considered an important etiological cause of ASD and may be responsible for impairments in social conduct [45]. To test this hypothesis in the valproate and fragile X rodent models of autism, researchers abolished the neuroprotective, oxytocin-mediated GABA excitatory-inhibitory shift during delivery [45]. The newest studies on the central OXT system in VPA-induced rat models of autism confirm this hypothesis. A lower level of OXT was measured in the hypothalamus mRNA of adolescent VPA rats and the supraoptic nucleus (SON) of neonatal VPA rats [46]. Both these groups exhibited autistic-like behaviors. Intranasal administration of OXT restored the social preference of adolescent VPA rats. Early postnatal OXT treatment had a long-term therapeutic influence on autistic-like behaviors in VPA rats [46].

Investigations in animal models of ASD are consistent with similar findings in humans. In addition to being well-tolerated by humans, oxytocin significantly improves social abilities and behaviors in children with ASD [47].

One double-blind, randomized, placebo-controlled trial administered intranasal OXT for four weeks to 40 adult ASD patients [48]. Results showed no treatment-specific improvements but did indicate a positive trend in secondary outcomes such as repetitive behaviors and feelings of avoidance. This trial concludes by suggesting multiple-dose intranasal OXT regimen studies to assess its long-term efficacy [48].

Another study aimed to elucidate the treatment mechanism of intranasal oxytocin (IN-OXT) in 38 adult ASD patients and address the previous study’s suggestion [49]. As an endogenous neuropeptide, oxytocin has a neuromodulatory role in governing social behavior. Levels of OXT can influence cooperative and affiliative behavior, interpersonal attachment, and bonding. Anatomically, OXT action was assessed by imaging the participants' amygdalae and posterior superior temporal sulci (pSTS). Reduced bilateral amygdala activity is associated with improved social behaviors, while the inverse stands true for pSTS activity [49]. Participant behavior was assessed by measuring IN-OXT action in two emotional states: happiness and anger.

Initially, a single dose of IN-OXT showed different effects on amygdala activity in other emotional states. Apparent attenuation was observed in happy states, while the opposite was true for angry states. However, multiple doses of IN-OXT eventually reduced bilateral amygdala activity in both emotional states compared to the placebo-controlled group. These results indicate that multiple-dose oxytocin treatment provides long-term improvement of social behavior through reliable attenuation of amygdala activity regardless of hemisphere or emotional state (happy *vs.* angry).

In contrast, a single dose of IN-OXT showed stark improvements in social behavior related to amplifying pSTS activity regardless of hemisphere or emotional state. However, a generalized negative trend was observed in multiple-dose regimens. It is interesting to note that the right pSTS showed an overall better response to multiple-dose IN-OXT therapy compared to the left pSTS.

Consequently, the study recommends multiple-dose IN-OXT treatment regimens intending to induce neural changes in ASD patients that outlast those resulting from four-week regimens [49].

## 4 MITOCHONDRIAL DYSFUNCTION IMPROVEMENT

### 4.1 Antioxidant Therapy

Recent studies about the pathophysiology of ASD revealed oxidative stress and inflammation to have a role in its development [50, 51]. Results showed reduced endogenous antioxidant enzyme levels and increased oxidative stress biomarkers in autistic subjects. As such, it was suggested that antioxidant compounds could be used as viable therapeutic tools to minimize, or ideally prevent, the changes caused by free radical damage in patients who have ASD [52, 53]. Sulforaphane is one of the antioxidants studied intensively in ASD patients. It is contained in such vegetables as broccoli, cauliflower, cabbage, *etc.* According to the results from double-blind, randomized, placebo-controlled clinical trials, sulforaphane significantly improves core behavioral and cognitive symptoms among ASD patients [54].

### 4.2 L-Carnosine Supplementation

A meta-analysis of four double-blind placebo-controlled RCTs and one open-label trial was aimed to assess l-carnosine’s proposed neuroprotective, antioxidant, and anti-convulsive effects [55]. However, the results were inconclusive due to a lack of well-designed RCTs with bigger sample sizes [55].

One study aimed to test the efficacy of L-Carnosine as an add-on to risperidone in 70 children with ASD [56]. It concluded that L-Carnosine treatment did not notably change irritability subscale scores but did improve the hyperactivity/noncompliance subscales of the Aberrant Behavior Checklist-Community (ABC-C) rating scale in patients with ASD [56].

Also, a meta-analysis of three studies revealed no noteworthy differences between experimental L- Carnosine-supplemented and placebo-controlled groups on the Gilliam autism rating scale [55].

### 4.3 L-Carnitine Supplementation

Carnitine participates in a shuttle system, which transports long-chain fatty acids into mitochondria for oxidation and energy production. Some treatments for mitochondrial disease are found to improve core and associated ASD symptoms. Some ASD patients have disorders in L-carnitine synthesis (around 10%-20%), and supplementation is the treatment of choice [57].

One study divided 30 children with ASD into experimental L-Carnitine-supplemented and placebo-controlled groups. The experimental group showed significantly improved CARS scores overall, with statistically significant differences in free and total carnitine levels but no established link between the former and the latter. Overall, the study recommends a 6-month course of L-Carnitine supplementation to improve autism severity but also suggests further studies to concur with this recommendation [58].

Systemic PCD is associated with many adverse clinical findings, including, but not limited to, hepatomegaly, muscle weakness, elevated transaminase levels, hyperammonemia, and altered gastrointestinal motility [59]. Neurodevelopmental disorders, including ASD, have yet to be reliably linked to PCD. However, one case report examined a 7-year-old girl with ASD secondary to Systemic Primary Carnitine Deficiency (PCD) [59]. This report identified a PCD gene mutation in a young girl with ASD. The report suggests that routine PCD screening in infants might be able to identify carnitine deficiency early on and prompt early supplementation to prevent brain carnitine deficiency that manifests as functional alterations later on. This young girl was diagnosed with PCD later than usual, and the report attributes this delay to the expressed severity of her phenotype. Furthermore, the report believes that delayed diagnosis of PCD and carnitine supplementation played a part in the patient’s only mild to moderate response to treatment, where changes in ASD features were not observed [59]. Overall, the possible emerging link between undiagnosed, progressively worsening PCD and subsequent functional alterations in the brain, which may manifest as ASD, needs to be explored further by subsequent studies.

Recently, there have been studies that describe the efficiency of combined therapy. A randomized double-blinded placebo-controlled clinical trial showed improved ASD symptoms (social isolation, stereotypic behavior, and inappropriate speech) after adding l-carnitine to risperidone in treating children and adolescents with ASD [60].

### 4.4 Butyrate Treatment

Butyrate is one of the major metabolites of the gut microbiome. It is produced primarily by anaerobic microbes such as Clostridium clusters IV and XIVa [61]. Although Lactobacilli do not actively produce BTA, they contribute to the growth of clostridia that do so [62]. Butyrate is responsible for many regulatory activities, some of which include, but are not limited to, improving mitochondrial function during oxidative stress, regulating the integrity of gut barrier functions and, consequently, the microbiome-gut-brain axis [63], utilizing its anti-inflammatory potential to fortify mucosal immunity, and increasing rates of expression in genes thought to be linked to cognition and behavior (CREB1, CamKinase II).

In addition, some studies report data that show butyrate treatment leading to improvements in ASD test subjects, be it regulation of social behavior in autistic mice [64], acting on specific transporters and receptors to mitigate aberrations in the gut-brain axis [65], or suppressing Histone Deacetylase activity, thereby improving immune response [63].

### 4.5 Resveratrol Treatment

Resveratrol (RSV) is an anti-inflammatory and antioxidant compound that indirectly enhances mitochondrial function and prevents social impairments in the VPA animal model of autism [66]. During prenatal exposure of animals to VPA, RSV has preventive effects on changes in sensory behavior, the localization of GABA-ergic parvalbumin (PV+) neurons in sensory brain regions, and the expression of proteins of excitatory and inhibitory synapses. Treatment with RSV prevents all the pathologic changes in the brains of experimental animals triggered by VPA [67]. The role of RSV in regulating mitochondrial fatty acid oxidation (mt-FAO) and energy homeostasis is also being examined as a possible treatment strategy for ASD [66].

### 4.6 Rapamycin Therapy

The mammalian target of the rapamycin (mTOR) signaling pathway regulates cell growth and metabolism and is closely linked with intracellular oxidative stress when overactivated [68, 69]. Mice with tuberous sclerosis complex (TSC) were treated with rapamycin, a specific mTOR inhibitor, and recovered from deficits in social interaction [70]. Thus, regulating mitochondrial dysfunction with antioxidants could improve ASD behavior by regulating the mTOR pathway.

## 5 REGULATION OF METHYLATION. ADMINISTRATION OF VITAMINS B12 AND B9

**Fig. (2):**
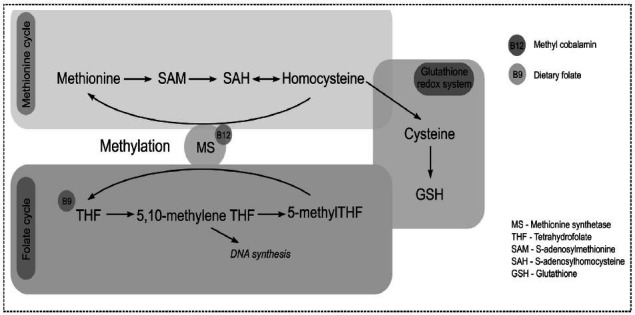
Glutathione regulation *via* methionine and folate cycles. Methionine and folate cycles are involved in synthesizing glutathione (GSH). All three metabolic pathways are interconnected, and the defect in some units (*e.g.*, SAM, SAH, cysteine, GSH) of the chain can be found in many patients with ASD. Methionine synthetase is the linking molecule between methionine and folate cycles. Vitamin B12 and 5-methyl-tetrahydrofolate are required for methylation by converting homocysteine into methionine and S-adenosyl methionine (SAM). Both processes contribute to the synthesis of GSH, the key player in redox homeostasis.

It is known that some key metabolic pathways involved in redox regulation are affected in patients diagnosed with ASD. Glutathione (GSH) is the main redox buffer produced in all cells [71]. Its production of it is highly connected to the methylation (B12) and folate (B9) metabolisms (Fig. **2**).

ASD patients have been found to have vitamin B12 deficiency [72-74]. Low levels of B12 lead to increased homocysteine levels and decreased S-Adenosylmethionine (SAM) levels. The latter is a common co-substrate involved in methyl group transfers, which results in DNA and histone methylation throughout the body, including brain tissue. Depleted SAM will decrease the GSH level and lead to a consequent suppression of antioxidant defense.

A clinical trial revealed that methyl B12 supplementation improved symptoms and reduced oxidative stress in autistic children [75]. Another study further elaborated on altered cobalamin levels by establishing a link between low levels of the antioxidant GSH to both ASD and schizophrenia and observed that both total cobalamin (vitamin B12) and methyl-cobalamin (active vitamin B12, methyl B12) levels were decreased in glutamate-cysteine ligase modulatory subunit knock- out (GCLM-KO) mice, which exhibited low GSH levels [76].

Folinic acid (vitamin B9) prevents behavioral deficits in rats as it is an analog of folate, which can pass through the blood-brain barrier even in the presence of the folate receptor (FRα) autoantibody [76]. These autoantibodies reliably block folate activity in children with ASD and their mothers, where they are found in excess concentrations [77]. Folic acid-mediated improvements in ASD and schizophrenia-associated behaviors are also observed in human patients. Folate supplementation during the preconception and gestational periods may prevent the development of ASD by enabling methylation of B12, activation of the methionine cycle, and production of the antioxidant GSH. Oral low-dose folic acid administered with subcutaneously injected methyl-cobalamine increased blood plasma levels of glutathione and were theorized to lead to increased antioxidant capacity and reduced oxidative stress in a subgroup of children with autism [75, 78].

Regarding the behavioral aspect of folic acid supplementation, a two-part study in China concluded that folic acid treatment increased homocysteine concentrations and regulated glutathione metabolism [79]. These improved biochemical pathways manifested as improved sociability, verbal and non-verbal language, and overall communication with others in the folate-supplemented children compared to the control group [80]. Meanwhile, a previous review of articles tackling the issue of folic acid supplementation in pregnant women increases the risk of ASD in their children concluded that results were contradictory mainly due to the inconsistency of study methods. As such, the review could not reliably verify that folic acid supplementation could increase the chances of ASD in unborn children [81].

## 6 RESTORING GUT MICROBIOTA

Alterations in gut microbiomes are possible pathways of ASD pathogenesis. Research is increasingly able to characterize the “fragile gut” in children with ASD, as evidenced by low digestive enzyme activity and impaired gut barrier integrity. These findings support the hypothesis that the entry of dietary peptides and metabolites from microbial activity from the gut lumen into the vasculature is associated with an aberrant immune response [82-84]. Regulation of “fragile gut” with dietary manipulations, such as microbiota transfer therapy, pre/probiotic therapy, fatty acid supplementation, and gluten-free/casein-free and ketogenic diets, have generally demonstrated improved GI and associated behavioral symptoms.

### 6.1 Microbiota Transfer Therapy

An open-label clinical trial assessed the influence of Microbiota Transfer Therapy (MTT) on gut microbiota composition and GI and ASD symptoms [82]. A nearly 80% decrease in GI symptoms was recorded at the end. These GI improvements persisted for eight weeks. In addition, behavioral ASD symptoms improved consistently over eight weeks and remained so after treatment was ended. After treatment, these patients' bacterial and phage deep sequencing analyses revealed successful partial engraftment of donor microbiota and beneficial alternations in the gut environment [82].

This trial indicates that MTT seems to be an excellent approach to improving ASD and comorbid GI symptoms by modifying the gut microbiome [82]. Hence, targeting the gut microbiome through pre/probiotic treatment, fecal microbiota transplantation, and biofilm eradication seems promising methods for ASD treatment [85-87].

### 6.2 Probiotics and Prebiotics

Probiotics, also known as “good bacteria,” reside in the gut and aid with metabolism, gut immunity, and health. Through their actions, these beneficial microorganisms regulate several neuro-intestinal processes through the so-called gut-brain axis. Prebiotics, like non-digestible carbohydrates, can be described as the food source for probiotics [88]. The implied synergistic correlation between probiotics and prebiotics is already apparent through their definitions alone.

In patients with neuropsychiatric conditions, such as ASD, numerous alterations in gut microbiomes have been observed [89]. Some populations, like Alistipes and Parabacteroides, were shown to be decreased, while others, like Corynebacterium and Lactobacillus, were increased [89]. These disbalances would ultimately manifest as common GI symptoms such as constipation, abdominal pain, diarrhea, and flatulence. Increasing evidence suggests that GI symptoms are common comorbidities in ASD patients [84]. Animal clinical trials have further reinforced cross-links between the two organ systems, showing that probiotics also have a lasting booster effect on the secretion of neuroactive agents like GABA and serotonin [88, 90]. A study in 50 young golden Syrian hamsters found that probiotics containing Bifidobacteria and Lactobacilli restored normal gut microbiota, decreased glutamate, and increased GABA and Mg^2+^ levels, indicating their potential as a safe treatment for glutamate excitotoxicity [91]. Also, probiotics have been shown to downregulate the HPA axis in depression cases [92].

Given the previous positive outcomes, several researchers have tried restoring gut microbiota in ASD patients to observe if it also affects neurobehavioral symptomatology. A 12-week randomized, double-blind controlled trial reports some improvements in GI symptoms but no changes in adaptive and repetitive behaviors [93]. A second randomized, double-blind, placebo-controlled study showed an inverse picture when prebiotic monotherapy and combination therapy were tested parallel. The study reports that monotherapy showed no GI improvements, whereas combination therapy, supplemented with gluten and casein-free diets, exhibited reduced anti-sociability scores [94]. A recent study investigated the effects of probiotics on 40 Egyptian ASD children aged 2-5. After three months of daily supplementation, there was a significant increase in Bifidobacterium and Lactobacillus spp. in the stool samples of the children. 80% of the children also showed reduced anxiety and improved sleep patterns [95].

A crossover-design pilot study supplemented children with ASD and chronic GI symptoms with bovine colostrum product (BCP) and a combination of BCP and B. Infants with a washout period between regimens [96, 97]. The researchers reported significant improvements in GI symptoms and stool quality alongside significant reductions in aberrant behaviors. However, given the numerous limitations of this study, namely the absence of a control group, the short duration of the research, and the continuation of concurrent ASD treatment plans in their participants, the authors encourage more robust studies to be carried out to discern the reliability of their findings.

### 6.3 Propionic Acid (PPA) Corrections through Biofilm Treatment

Propionic acid, as well as other enteric short-chain fatty acids, is detected in high quantities among human autistic groups and is linked to behavioral deficits owing to its ability to readily access the brain [98]. PPA administration in rats induces abnormal neural cell organization and numerous electrophysiological, behavioral, and neuropathological changes, typical for ASD (Choi *et al.* 2018) [99]. A lot of the detrimental effects of PPA can be attributed to its concentration in the body. Propionic acid is produced by gut bacteria, especially Clostridia, which produce PPA and exotoxins [100]. Interestingly enough, children with ASD have been found to have gut microflora different from neurotypical ones [101, 102]. These differences are mainly related to the sizes of other bacterial populations. An imbalance is evident in such people, where levels of Bacteroidetes are decreased, but those of fungal Candida are increased [89]. Ultimately, these imbalances break the intricate cycle of production and degradation of PPA between these populations, and the resultant build-up of PPA concentrations is observed, causing behavioral impairments.

Several clinicians who follow clinical protocols for biofilm eradication therapy have been able to balance PPA concentrations. This protocol is a step-wise, nutrition-based approach to restoring the integrity of the human gut microbiome. At first, proteolytic enzymes like nattokinase, streptokinase, and other mucolytics are given to tear down the biofilm, exposing the underlying microbial colonies [103, 104]. Secondly, antimicrobial therapy is started with antibiotics and antifungals to destroy the dense populations. Then, binders like EDTA, Chitosan, and citrus pectin are used to clean up the remnants [105]. Finally, probiotics and prebiotics restore normal physiologic environments [106].

## 7 PROPOSED DIETARY CORRECTIONS

### 7.1 Gluten-free and/or Casein-free (GFCF) Dietary Intervention in Children with ASD

In 1979, Panksepp J. described similarities between low doses of narcotics and major symptoms typical for children with ASD [107]. He proposed that autism may be caused by endogenous overactivity of the child's own brain opiate system and called this phenomenon the “opioid excess theory” of autism. Afterward, the hypothesis was supported by several studies proving elevated levels of endorphin fractions in CSF [108] and beta-endorphin levels in peripheral blood mononuclears [109]. The important premise of “opioid excess theory” proposes that some nutritional proteins like gluten from wheat and casein from milk can be risks for ASD development. Both gluten and casein eventually yield peptides called gluteomorphine (or gliadorphin) and casomorphins, respectively [110]. These peptides can bind to opiate receptors in the CNS and mimic the effects of opioids. Thus, it can be speculated that these peptides, formed during digestion, lead to up-regulation of the endogenous opioid system, which is linked to the symptoms of autism.

The other “update” on “opioid excess theory” describes that gluten and casein might be metabolized insufficiently. Considering that children with ASD have a “leaky gut,” these two compounds pass through a very thin intestinal membrane to penetrate the BBB and, thereby, the CNS, resulting in aberrant neurotransmission [111]. So, the GFCF’s benefits rely on the “classical opioid excess theory” and its “update.”

Patients with celiac disease are sensitive to gluten proteins, which constitute the majority of the protein composition of wheat. Their intestinal T cells react to these gluten proteins by producing large quantities of interferon-γ [112]. This increased autoimmune response disables the digestion of gluten proteins and damages the surrounding intestinal epithelium [113]. And, of course, we should not forget that casein protein and gluten are common allergens that elicit targeted IgA and IgG production [114].

Physicians are frequently being questioned about the GFCF diet as an alternative method of ASD management. However, research results still need to be conclusive. Some studies show that, according to the parents of their participants, children with ASD and comorbid GI symptoms show a greater improvement in ASD mannerisms than children with no comorbid GI symptoms [115]. Other studies claim that, although reported by the parents in their studies, there are no statistically significant changes between experimental and control groups [116, 117]. Finally, some studies denounce the majority of findings related to the GFCF diet as “seriously flawed” and recommend adherence only in case of food allergies or intolerance to gluten or casein is diagnosed [118].

### 7.2 Ketogenic Diet

A behavioral assessment of the effects of the ketogenic diet (KD) in ASD models shows positive changes in social behavior, such as higher sociability and social novelty indexes [119, 120]. Recent data indicate that a low-carbohydrate, moderate protein, high-fat diet significantly improves core autism features in animals as well as in humans, which are maintained for long periods [121-124].

One case-control study divided its participants into three groups [122]. The first group adhered to a modified ketogenic diet called the Modified Atkins Diet (MAD), which is considered less restrictive than the standard KD. The second group of participants was prescribed the GFCF diet. Finally, group 3 was reserved for control cases. All participants were assessed with the Childhood Autism Rating Scale (CARS) and the Autism Treatment Evaluation Test questionnaire (ATEC). The study concluded that both MAD and GFCF diets yielded decreased scores in CARS and ATEC, which correlate to improved scores overall [122]. Improvements were statistically significant in the majority of the studied parameters. Patients on the MAD diet showed significant improvements in CARS and ATEC scores, specifically in the speech, social, and cognition subsets (I, II, III) of the ATEC.

The study concluded that both diets had been proven to show improvements in participants with ASD, although these changes focused on different behavioral aspects depending on the diet [122]. Furthermore, participants in the GFCF group likewise showed statistically significant improvements in both assessment scores and, in particular, improved speech and behavior subset scores (I, IV) of the ATEC. However, they could not recommend a clear favorite diet based on their results. The study continued to cite a plethora of literature supporting and disproving their results. Further on, the authors referred to the limitations of their research and recommended larger-scale replications to reliably reach a verdict regarding the therapeutic efficacy of these two diets in patients with ASD.

A six-month pilot study included 30 participants between the ages of 4 and 10 who opted for the John Radcliffe keto diet [123]. A total of 18 participants completed the course with varying levels of psychosomatic improvements. The authors mentioned that autistic children have deficient glucose oxidation and postulated that ketone bodies were an adequate alternative fuel for their brains. Finally, the authors cited some limitations that interfered with the formulation of further hypotheses. The first was the heterogeneity of their participants regarding different subgroups of biochemical responses to their interventions. The second was the difficulty of some children, especially those with more severe cases of ASD, to adhere to the dietetic plan. The last one was the uncertainty surrounding the optimal duration and manner in which the diet could be applied to their participants.

## 8 CHELATION THERAPY

Chelation therapy is a medical treatment that utilizes chelating agents to remove toxic metals from the body [125-127]. However, its use in children with ASD has been a topic of debate due to mixed results from studies. Choosing the appropriate chelating agent depends on factors such as the type and severity of metal toxicity and the individual's overall health status. In one clinical study, meso-2,3-dimercaptosuccinic acid (DMSA) effectively reduced levels of lead and mercury (Hg) and improved autistic symptoms in children with high levels of these metals [128].

Metallothioneins (MTs) are small proteins that bind to metals and prevent harmful effects, but toxic metals such as Hg can displace Zn and Cu from MTs, potentially causing negative effects. Chelation therapy increases the urinary excretion of the essential trace elements Cu and Zn and may lead to deficiency symptoms, making monitoring of Cu and Zn levels crucial. Excessive levels of Cu in the body can interfere with Zn absorption, affecting the transcription of MT genes and eliminating toxic metals. This highlights the importance of understanding toxic metals and patients’ health [17].

Chelation therapy must be performed under medical supervision after carefully considering its potential risks and benefits. A data review identified serious adverse events associated with chelation therapy in ASD, such as hypocalcemia, renal impairment, and reported deaths, concluding that the risks currently outweigh any proven benefits [129].

## 9 ANTI-INFLAMMATORIES

### 9.1 Non-steroidal Anti-inflammatories

Non-steroidal anti-inflammatory drugs, *e.g.*, Ibuprofen (non-selective COX-1 and COX-2 inhibitor) and Acetaminophen (COX-3 inhibitor), were studied as anti-inflammatory agents during fever in children with ASD. Not only was Ibuprofen observed to have less association with ASD development in children than Acetaminophen, but usage of the latter in children aged 12 to 18 months also made them eight times more likely to have ASD than control children [130].

### 9.2 Vitamin D Supplementation

Among all vitamins implicated in ASD pathology, none correlate quite as strongly and quite as researched as Vitamin D. Several studies linked Vitamin D deficiency in pregnant mothers to children born with ASD [131, 132]. Consequently, research was targeted at establishing a connection between low levels of Vitamin D and ASD and the pathways by which Vitamin D supplementation led to the suppression of ASD-associated symptoms. The anti-inflammatory effects of Vitamin D are manifested by how well it can antagonize inflammatory cascades induced by Lipopolysaccharide (LPS) through inhibiting MAPK pathways and the consequent production of inflammatory molecules [133]. Vitamin D is also documented to have a synergistic effect with Serotonin. Calcitriol activates Tryptophan Hydroxylase 2 and inhibits Tryptophan Hydroxylase 1 transcription, increasing serotonin in the brain, ultimately translating to prosocial behaviors. This observation is absent in anti-social, autistic children [134]. To date, considerations for Vitamin D supplementation were backed by a single randomized control trial that showed increased retention of its precursor, 25(OH)D, in children with ASD, showed significantly better scores across all tested behavioral measuring checklists after four months of supplementation [135].

### 9.3 Hyperbaric Chamber Therapy Option

One of the emerging methods of ASD treatment is hyperbaric oxygen therapy (HBOT) through its anti-inflammatory effects in children with cerebral hypoperfusion. Patients who need hyperbaric oxygen therapy (HBOT) are placed in a chamber filled with 100% oxygen gas at a pressure greater than one atmosphere absolute. The benefits of this therapy are faster tissue recovery and improved physiological aspects due to an increased oxygen supply.

Previous neuroimaging research detected cerebral hypoperfusion in children with ASD [136]. HBOT counteracts cerebral hypoperfusion in children suffering from arterial insufficiency, air or gas embolism, gas gangrene, carbon monoxide poisoning, and decompression sickness.

One study states that HBOT inhibits underlying chronic opportunistic infections in children with autistic disorders. However, case series and randomized controlled trials so far don’t show any evidence to support this theory. Only a single multicenter, randomized, double-blind, controlled trial found positive changes in Aberrant Behavior Checklist-Community (ABC), Autism Treatment Evaluation Checklist (ATEC), and Clinical Global Impression-Improvement (CGI) scores [137]. Another randomized, double-blind, placebo-controlled trial detected no differences in social reciprocity, communicative approach, and repetitive behaviors between HBOT and placebo groups [138]. These results have yet to be repeated.

Some researchers describe HBOT as a dosing agent involving two variables: oxygen and pressure [138]. Positive effects are more likely to be detected when a delicate balance unique to each individual is achieved during testing. However, this ambitious goal still needs to be eradicated on a grand scale. Consequently, numerous authors still refrain from publishing final verdicts on the efficacy of HBOT in patients with ASD, always mentioning that further targeted, large-scale research needs to be conducted in the future. The FDA maintains its stance against encouraging HBOT in children with ASD due to the lack of conclusive evidence for the latter’s symptomatic treatment.

## 10 MINERAL IMBALANCES

### 10.1 Zinc and Copper

Zinc is an essential trace element crucial in maintaining optimal brain function [139, 140]. Its deficiency can lead to various neuropsychological changes, including emotional instability, irritability, and depression [141, 142]. Zinc is also essential for cognitive performance and is involved in glutamatergic transmission, which has short-term and long-term mental effects [17]. Zinc blocks NMDA receptors and is an intracellular signal factor involved in numerous proteins, and its deficiency may impair neurotransmission [143]. Furthermore, Zn acts as a co-transmitter with glutamate, preventing excitotoxicity, and is an important trophic factor, providing essential nutrients for enzymes with anabolic function in synapse areas undergoing permanent learning processes [17].

Copper toxicity can severely affect the brain and lead to symptoms such as depression, irritability, fear, nervousness, learning, and behavioral problems [17, 142]. Copper is a cofactor required for dopamine-β-hydroxylase (DBH) activity, which converts dopamine to norepinephrine [144]. Excess Cu can increase norepinephrine levels, which have been found in individuals with ASD. Moreover, Cu inhibits the enzyme hydroxytryptophan decarboxylase, leading to decreased production of the neurotransmitter serotonin. Hypercupremia, caused by excess copper, may be related to depression. Too much Cu/Zn-dependent superoxide dismutase may also increase oxidative stress, leading to redox imbalance due to reactions with hydrogen peroxide (H_2_O_2_) and peroxynitrite [17].

The trace elements Zn and Cu are inversely related, and their levels in the body are critical for maintaining various biological functions. Cytokines help maintain this balance by enhancing the cellular uptake of Zn and the production of ceruloplasmin in the liver. The normal ratio of Zn to Cu in neurotypical children and adults is close to 1:1 [17]. The plasma zinc/serum copper ratio can quickly determine the functional state of MTs. Studies have found that children with ASD often have lower Zn/Cu ratios than neurotypical children, which may indicate total body Zn deficiency or accumulation of Zn-antagonistic toxic metals [17, 141, 142]. Mercury toxicity may significantly contribute to MT dysfunction in children with ASD, which may also be reflected in the Zn/Cu ratio [17].

Toxic metals such as cadmium (Cd) and Hg may have opposite effects on Zn and Cu metabolism. Enhanced MT induction in the liver may affect Cu excretion *via* the bile more than its mobilization from the liver to the blood. At the same time, for Zn, the mobilization rate to the blood is more strongly affected. Increased MT induction due to oxidative stress may also lead to enhanced retention of toxic metals such as Cd and Hg in organs like the liver and kidney, which could explain higher levels of these metals in ASD patients. However, once toxic metals accumulate, they can interfere with Zn metabolism, further exacerbating the patient's condition [17].

ASD patients may have less tolerance to toxic metal exposure, and enhanced MT induction due to oxidative stress may interfere with the regulation of Cu excretion through bile, leading to abnormal accumulation in the liver and potentially causing Cu toxicity. This accumulation may interfere with Zn metabolism and decrease tolerance to toxic metal exposure. Enhanced apometallothionein induction due to oxidative stress may have opposite effects on Cu and Zn metabolism, simultaneously enhancing the severity of Zn deficiency and Cu toxicity [17].

### 10.2 Selenium

Several studies have suggested that disturbances in selenium (Se) metabolism may be involved in the pathogenesis of ASD [145, 146]. Oxidative stress, commonly observed in ASD, has been linked to mitochondrial dysfunction, immune dysfunction, and inflammation [50, 51, 146]. Selenium has been shown to alleviate these conditions, and as a result, several studies have investigated its potential role in ASD [147, 148]. However, the exact mechanism remains unclear, as some studies have reported altered expression of selenoproteins in ASD, while others have not identified significant changes [147].

Selenium may protect against the harmful effects of Hg [149, 150]. It has been suggested that Se may also protect against heavy metal toxicity, which has been hypothesized to play a causative role in ASD development [151]. The complex interaction between selenium and Hg may have clinical implications in ASD and other neurological disorders [149]. However, the nature of this interaction and its potential role in ASD pathogenesis and treatment requires further investigation.

ASD has been linked to alterations in gut microbiota, and Se deficiency has been shown to result in changes in bacterial populations associated with lipopolysaccharide (LPS) overproduction and translocation. Selenium may modify LPS-induced inflammatory pathways by protecting against endotoxemia through modulation of p38 MAPK and NF-κB [147].

While the evidence regarding the potential role of Se disturbance in ASD is limited and conflicting, Se is an essential micronutrient that may offer potential benefits in alleviating some of the symptoms of ASD. Further research is needed to elucidate the exact mechanisms by which Se may exert its effects on ASD and to determine the optimal dose and duration of supplementation required to achieve the maximum benefit. Understanding the role of Se in the pathogenesis of ASD may provide new insights into developing effective treatment strategies.

### 10.3 Calcium and Magnesium

Calcium (Ca) and magnesium (Mg) are essential minerals that play crucial roles in the body's functions, including neurological development and function [152]. Research has suggested deficiencies or imbalances in these minerals may be involved in the pathogenesis of ASD [153].

Calcium is the most abundant mineral in the body and is essential for various functions, including muscle contraction, blood clotting, and nerve function [154]. It also plays a vital role in regulating the communication between neurons, which is necessary for proper brain function [154]. Studies have suggested that Ca dysregulation may affect ASD development [155]. Research has shown that ASD children had lower serum Ca levels than neurotypical children [156]. Calcium dysregulation has been linked to oxidative stress, inflammation, and mitochondrial dysfunction, commonly observed in ASD individuals [155]. Calcium channel blockers, which regulate Ca influx into cells, improved social behavior in mice ASD models.

Magnesium plays a vital role in the body's functions, including muscle and nerve function, energy production, and protein synthesis. Magnesium also plays a crucial role in regulating Ca levels in the body [157]. Magnesium dysregulation has been linked to oxidative stress, inflammation, and mitochondrial dysfunction, commonly observed in individuals with ASD [158]. In some studies, ASD children had lower serum Mg levels than neurotypical children [159, 160].

The interaction between Ca and Mg is essential for proper body function. Magnesium regulates Ca body levels by activating vitamin D, which is necessary for Ca absorption in the intestines [161]. Magnesium regulates Ca influx into cells, which is essential for proper neurological function. Magnesium inhibits glutamate release, an excitatory neurotransmitter that can lead to Ca influx and oxidative stress [162]. Research indicates that the Ca-to-Mg ratio may be involved in ASD pathogenesis. In a study, ASD children had a higher Ca-to-Mg ratio than neurotypical children. In another study, the Ca-to-Mg ratio positively correlated with autism severity. Research also indicates that children with both ASD and ADHD exhibit more significant changes in their hair and urinary Mg levels compared to neurotypical children [160]. Magnesium may protect against the harmful effects of Ca dysregulation. Magnesium also has anti-inflammatory properties and may protect against mitochondrial dysfunction, commonly observed in children with ASD.

The role of Ca and Mg in ASD is complex, and further research is needed to elucidate their exact mechanisms. However, research indicates that Ca and Mg dysregulation impacts ASD pathogenesis [163]. Supplementation with Ca and Mg may alleviate some of the symptoms of ASD [164]. However, the optimal dose and duration of supplementation required to achieve the maximum benefit still need to be determined.

## 11 VITAMINS

Vitamin supplementation is a common adjunct therapy used in individuals with ASD due to the links between certain vitamins and ASD pathology [165-167]. Vitamins A, C, and E are known to boost antioxidant capabilities, while vitamin D has anti-inflammatory and serotoninergic roles [134, 168, 169]. Vitamin B1 is crucial for energy regulation [170], and vitamin B6 regulates excitation and inhibition balance through neurotransmitter synthesis, particularly GABA regulation [171, 172]. Vitamins B9 and B12 play a role in methylation regulation [173].

Individuals with ASD often have metabolic and nutritional abnormalities, including sulfation, methylation, glutathione redox imbalances, oxidative stress, and mitochondrial dysfunction [174-176]. Vitamin supplementation may support these physiological processes. Non-randomized double-blind study where 16 ASD children were enrolled and received high doses of B6-Mg reported significant behavioral changes [177]. However, the role of B6 supplementation in ASD is still unclear. Vitamin D supplementation may also benefit individuals with ASD due to its immune function modulation and inflammation reduction properties [178].

It is important to note that optimal dosages and durations of vitamin supplementation in individuals with ASD are unclear. Moreover, vitamin supplementation may not be effective for all individuals with ASD and should be determined on a case-by-case basis [166].

## 12 TRYPTOPHAN

Tryptophan, an essential amino acid and precursor to serotonin, may benefit individuals with ASD by improving social behavior, decreasing repetitive behaviors, and increasing cognitive flexibility [179, 180]. Its mechanism of action in ASD is not fully understood, but it may increase brain serotonin levels and have anti-inflammatory effects. However, its use should be cautiously approached due to potential side effects and the risk of serotonin syndrome when combined with certain medications. While tryptophan may be deficient in individuals with ASD, supplementation with B vitamins and magnesium may influence tryptophan metabolic homeostasis [179]. Further research is needed to understand better the benefits and risks of tryptophan supplementation in ASD.

## 13 IMMUNOTHERAPY

Individuals with ASD often exhibit altered immune responses, including T cells, B cells, monocytes, natural killer cells, dendritic cells, and elevated cytokines and neuroinflammation. Immune dysregulation and inflammation are crucial components of ASD diagnosis and treatment [181]. Understanding immune dysregulation and inflammation in ASD can significantly advance diagnosis and treatment. Immunotherapy, such as intravenous immunoglobulins (IVIG) injection, has shown promise in improving symptoms [182]. However, further research is necessary to identify additional immune biomarkers and investigate long-term risks and effects.

## 14 POSSIBLE THERAPY WITH CANNABINOIDS

In 2018, the National Academies of Sciences, Engineering, and Medicine published a report that presented strong evidence that cannabis positively affected patients with multiple sclerosis, chronic pain, spasticity, nausea and vomiting due to chemotherapy, and individuals experiencing seizures. This report also highlighted the lack of evidence supporting medical cannabis use in ASD [183]. Although specific strains and variations of cannabinoids are approved and recommended for therapeutic use, there are currently no research-backed recommendations for ASD patients.

Also, current pharmacological treatments can only alleviate some ASD symptoms without addressing the underlying etiology [184]. Some researchers maintain that dysregulation of the endocannabinoid system may play an important role in ASD pathophysiology and may represent a target for pharmacological intervention [185]. As of September 2020, there are four ongoing clinical trials, two of which are randomized controlled trials, and the other two are open-label trials [186]. Researchers have observed that the two main compounds currently being studied are CBD and THC, along with their purified extracts. Studies on cannabis show that it enhances social communication and decreases small-group hostility [187]. The knowledge regarding the use of medical cannabis among children and adolescents is still very limited. The medical and scientific society requires more information to define the rationale for prescribing medical cannabis among children. The potential side effects and related risks should be observed very closely before the routine prescription of the compound.

## 15 A BRIEF LOOK AT CELLULAR THERAPIES

As the understanding of ASD expands, the complexity of its etiology becomes more apparent. Although rooted as a genetic condition, research shows that external cofactors like interactions with environmental influences also play a significant role in ASD pathogenesis. One of the plausible pathways of ASD pathogenesis is immune dysregulation, specifically prenatal exposure to maternal immune activation (MIA). One review quotes several epidemiological studies that found a correlation between maternal infection during pregnancy and an increased frequency of ASD in their children [188].

Studies show that MIA ultimately leads to ASD in offspring through an immune cascade where IL-6, maternal Th17 cells, and Il-17A are the main factors [189]. Th17 cells are differentiated under increased Il-6 action secondary to maternal infection and start to oversecrete Il-17A, which passes the placental barrier and starts to act on fetal neural cells that express the receptor Il-17RA. This receptor activation initiates a cascade of intracellular signaling pathways that disrupt developmental processes like neural stem cell (NSC) proliferation, blood-brain barrier activity, gliogenesis, microglial function, and neuronal connectivity. The disruptive effects of this cascade later manifest as behaviors typically associated with pediatric ASD.

Stem cells vary greatly by their function and origin. To study their links to ASD, researchers isolated specific subtypes of stem cells, each possessing unique abilities and viability for practical testing. They noticed that all stem cell types have the universal abilities of homing and mobility. The current subtypes of stem cells under study for ASD links include fetal stem cells (FSC), mesenchymal stem cells (MSC) [190], hematopoietic stem cells (HSC), and umbilical cord [191]. One subtype is considered the cutting-edge variant in stem cell therapy and is called induced pluripotent stem cells (iPSC).

Most subtypes above are currently being studied for viability in various scenarios. Study objectives are focused on their safety, cost efficiency, the efficacy of therapy, possible side effects, viability for long-term treatment, teratogenic effects, and ease of sourcing, among others. Two promising clinical trials showed positive outcomes in improving ASD symptomatology with minimal adverse effects. The first one, an open-label prospective study, focused on treatment with FSCs and reported improvements in mood, eye contact, and appetite among some patients [192]. The second one, a proof-of-concept study, focused on transplanting human cord blood mononuclear cells and umbilical cord-derived mesenchymal stem cells [193]. Dawson *et al.* conducted a clinical trial where twenty-five children with ASD diagnosis were enrolled. This study reported the safety of umbilical cord blood single infusion and some behavioral improvements assessed 6 and 12 months after treatment [194]. The same group conducted a randomized controlled study to evaluate the safety and efficacy of cord blood infusion for treating ASD. Results did not indicate significant changes in primary clinical outcomes (social communication, autism symptoms) between the groups six months after the single infusion [195].

The iPSC subtype is used to generate three-dimensional models of neurons and brain structures to study autism pathophysiology [196]. iPSCs, when derived from ASD patients, exhibit morphogenetic abnormalities during early development where young postmitotic neurons have smaller cell bodies, more extensively branched neurites, and reduced motility compared with controls [197]. Two separate studies examined iPSC-derived neurons from autistic subjects, and both reported aberrant expression of cation channels, voltage- gated currents, and changes in synaptic functions [198, 199]. Some reports say that the iPSC subtype poses an increased risk for teratoma generation due to residual pluripotent stem cells in grafting experiments and is aptly considered too risky to be used reliably in clinical trials [200].

Having only a few small-scale studies, it is still very early to project any definitive answers regarding stem cell therapy in ASD patients. The strict adherence to regulatory, ethical, and safety guidelines, as well as cellular characteristics like the absence of homogenous cell populations, stability, and long-term survival of neurons after transplantation, means the speed of progression of research in the field is markedly slower than desired.

## 16 SUGGESTIONS

During this paper's conceptualization, organization, and preparation, it was noticed that most therapeutic interventions for ASD-associated symptoms were seldom prescribed in combination. As such, the synergistic capacity of these individually positive methods is still unexplored. Based on available information, a timeline structure was decided where several of these methods either overlap or directly follow one another. Following such a structure may yield greater improvements in patient quality of life and satisfaction from their experienced medical care. The primary incentive behind this recommendation is to provide management with greater efficacy to autistic children as soon as possible. Special care was taken to recommend the safest methods as first-line therapy, which would imply less problematic side effects, reduced implementation costs, and greater acceptance by the parents of these children. This timeline is by no means, a complete guide. The authors of this paper would be delighted to be contacted by the scientific community to engage in dialogue and discussions on how to improve this plan for the betterment of the increasing pediatric population suffering from ASD.

**Fig. (3):**
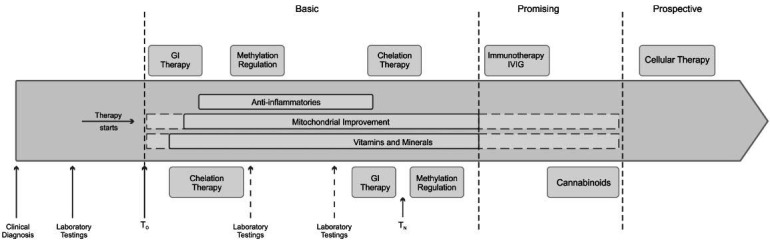
Suggested timeline for viable treatment approaches in ASD. A timeline starts with an accurate clinical diagnosis of ASD confirmed by lab tests, indicating the need to initiate treatment. Treatment begins at T_0_, with gastrointestinal imbalances being the first target (GI therapy). Vitamin/mineral supplementation, mitochondrial improvement, and anti-inflammatory methods are recommended as adjuncts throughout the entire regimen. All treatment approaches are divided into three broad categories depending on their clinical practicality. Basic/current methods are clinically safe and ready for usage. Promising methods are currently under study and are showing positive results. They may be clinically recommended soon and implemented in the chart accordingly after approval. Finally, prospective methods are still in their infancy, but researchers have high hopes that they will be able to provide far more radical and effective ASD treatment when tested sufficiently.

The suggested timeline (Fig. **3**) recommends organizing care into regular treatment regimens (N) after considering the prescribed medications' safety, practicality, invasiveness, and diet. Patients may receive several regimens (N=x) based on how well they improve after their first one (N=1). The basic screening for the targets that can be pharmacologically or dietary enhanced at the beginning of the treatment will allow the doctors to start the individualized scheme for each patient. Also, regular laboratory testing is recommended to assess patients’ conditions, follow-up treatment success, and to examine the possible toxicity status as a side effect of medications. Such frequent and meticulous follow-ups will allow clinicians to decide their next step accurately. They may restart the regimen after a certain period of watchful waiting or progress into the timeline into promising methods. A tailored approach to every case may be the best way to tackle this complex disorder. In turn, the suggested timeline for treatment approaches should be more personalized based on symptoms. The better the peculiarities of ASD are understood, the better to start constructing treatment approaches in the future.

## CONCLUSION

The etiology of ASD is unknown, but certain inborn errors of metabolism are shown to increase the risk of autistic behavior. At present, several genes are linked to the development of ASD. This disorder also involves environmental factors triggering physiological abnormalities in genetically sensitive individuals, so a complex of hereditary and environmental factors induces it.

The causal role of genetic mutations and environmental factors in the etiology of ASD can be investigated by preclinical tools provided by animal models. Knowledge about ASD and earlier recognition allows people with ASD and their families to receive early assistance for their condition.

Numerous preclinical studies on ASD mouse models contributed to the identification of new candidate drugs or other strategies to treat ASD in humans, including mGluR5 modulators, agmatine, rapamycin, oxytocin, supplementation with folate and vitamin B12, ketogenic diet, among others, thus confirming the relevance of this approach in translational ASD research.

The present review mostly discusses pharmacological approaches to manage the disease flow in a patient diagnosed with ASD. However, it is reasonable to believe that cognitive-behavioral treatment is important and must be initiated as soon as the diagnosis is established. Patients with affected social communication domains will benefit the most from this approach.

The way forward in ASD treatment should begin with standardizing and classifying treatment methods into a spectrum. By starting with GI therapy, chelation, and nutritional supplementation as first-line therapy, greater acceptance can be assured among the concerned demographics, especially in parents of children with ASD. A case-by-case approach should be established to better follow up on severe cases of ASD with increasingly targeted treatment methods like antimicrobials, antivirals, and immunotherapy. With advances in ASD research, it is reasonable to believe that stem cells hold the key to universal ASD resolution in the future.

## AUTHORS’ CONTRIBUTIONS

Conceptualization and design, K.Y.; Data Collection, K.Y., Z.O., M.M., L.H., N.Z., R.S., G.B.; Analysis and Interpretation of Data, K.Y., Z.O., M.M., L.H., N.Z., R.S., G.B.; Writing - Original Draft Preparation, K.Y., Z.O., M.M., L.H., N.Z., R.S., G.B.; Writing - Review and Editing, K.Y., Z.O., G.B.; Supervision, K.Y; Obtained Funding: K.Y. All authors have read and agreed to the published version of the manuscript.

## Figures and Tables

**Table 1:** Classification of suggested ASD treatment strategies in humans.

Current/basic strategies	Improvement of E/I Imbalance Risperidone Glutamate modulators NMDA-receptor modulators GABA-receptor agonists rTMS Oxytocin Improvement of Mitochondrial Dysfunction Antioxidants L-Carnosine L-Carnitine Butyrate Resveratrol Rapamycin Regulation of Methylation B12, B9 GI Regulation/Restoring Gut Microbiota Microbiota Transfer Therapy (MTT) Probiotics / Prebiotics PPA Corrections through Biofilm Treatment Dietary Corrections (GFCFD, KD) Chelation Therapy DMSA Anti-Inflammatory Treatment NSAIDS Vitamin D HBOT Vitamin and Mineral Supplementation Zink and Copper Selenium Magnesium and Calcium Vitamins A, C and E, vitamin D, vitamin B1, vitamin B6, vitamins B9 and B12 Tryptophan supplementation
Promising strategies	Immunotherapy IVIg Treatment with cannabinoids
Prospective strategies	Cellular therapy

## LIST OF ABBREVIATIONS

ASD: Autism Spectrum Disorde
CNVs: Copy Number Variants
ABC: Aberrant Behavior Checklist
CGI-S: Global Impression-Severity
CGI-I: Clinical Global Impression-Improvement
